# Decline in HIV Prevalence among Young Women in Zambia: National-Level Estimates of Trends Mask Geographical and Socio-Demographic Differences

**DOI:** 10.1371/journal.pone.0033652

**Published:** 2012-04-04

**Authors:** Nkomba Kayeyi, Knut Fylkesnes, Charles Michelo, Mpundu Makasa, Ingvild Sandøy

**Affiliations:** 1 Centre for International Health, University of Bergen, Bergen, Norway; 2 Department of Public Health, School of Medicine, University of Zambia, Lusaka, Zambia; 3 Ministry of Health, Lusaka, Zambia; Burnet Institute, Australia

## Abstract

**Background:**

A decline in HIV incidence has been reported in Zambia and a number of other sub-Saharan countries. The trend of HIV prevalence among young people is a good marker of HIV incidence. In this study, different data sources are used to examine geographical and sub-population group differentials in HIV prevalence trends among men and women aged 15–24 years in Zambia.

**Design and Methods:**

We analysed ANC data for women aged 15–24 years from 22 sentinel sites consistently covered in the period 1994–2008, and HIV data for young men and women aged 15–24 years from the ZDHS 2001/2 and 2007. In addition, we systematically reviewed peer-reviewed articles that have reported findings on HIV prevalence and incidence among young people.

**Findings:**

Overall trends of the ANC surveillance data indicated a substantial HIV prevalence decline among young women in both urban and rural areas. However, provincial declines differed substantially, i.e. between 10% and 68% among urban women, and from stability to 86% among rural women. Prevalence declines were steeper among those with the highest educational attainments than among the least educated. The ZDHS data indicated a significant reduction in prevalence between the two survey rounds among young women only. Provincial-level ZDHS changes were difficult to assess because the sample sizes were small. ANC-based trend patterns were consistent with those observed in PMTCT-based data (2002–2006), whereas population-based surveys in a selected urban community (1995–2003) suggested that the ANC-based data underestimated the prevalence declines in the general populations of both young both men and women.

**Conclusion:**

The overall HIV prevalence declined substantially among young women in Zambia and this is interpreted as indicating a decline in HIV incidence. It is noteworthy that overall national trends masked substantial differences by place and by educational attainment, demonstrating critical limitations in the current focus on overall country-level trends in epidemiological reports.

## Introduction

According to the 2011 report of The Joint United Nations Programme on HIV/AIDS (UNAIDS), a number of countries with generalised HIV epidemics, including Zambia, have experienced incidence declines since the year 2001 [1]. This conclusion is based on modelling of prevalence data from antenatal clinic sentinel surveillance (ANC) and national population-based surveys [2]. However, ANC-based sentinel surveillance has been and continues to be an instrumental data source for estimating HIV prevalence trends over time, although the extent to which these estimates are representative of the general population has been questioned [3]. Population-based survey designs are the gold standard in the provision of valid HIV estimates, but can be used as reference for validating ANC-based estimates only if biases due to non-participation or other sources have been carefully considered. Nationally representative demographic and health surveys (DHS) have included HIV testing in a number of sub-Saharan African countries. However, there are still too few DHS measurement points for trend estimation in most of these countries.

Although HIV incidence provides a more direct measure of the impact of HIV preventive and treatment programmes, it is more difficult to determine than prevalence [4]; cohort studies [5], [6] and biological assays that distinguish recent from established HIV infections [5], [7], [8] are very expensive and logistically difficult to conduct on more representative population samples [5]. Many studies have continued to use HIV prevalence among young people aged 15–24 years as a marker or indicator for HIV incidence [9], [10]. This age group has been found appropriate for estimating HIV incidence because it is less affected by AIDS-related mortality [11], [12] and people in this age group are assumed to have engaged recently in sexual debut.

Both ANC-based data and population-based data from Zambia have revealed great differentials in the magnitude and trends of HIV by place and socio-economic status [13], [14], [15], [16]. Accordingly, trend data at provincial and sub-group levels are critical for providing the details necessary for proper monitoring, evaluation and programming. This article examines and compares data from the national ANC-based HIV surveillance system (1994–2008) and two rounds of the nationally representative population-based Zambia Demographic and Health Survey (ZDHS) (2001/2–2007) by urban-rural residence, province, age-group and education among young people (15–24 years). Several other studies on HIV prevalence have been conducted in Zambia, and this paper also provides a review of published peer-reviewed articles to compare the ANC- and DHS-derived HIV prevalence and incidence estimates with estimates from other data sources.

## Methods

### National ANC-based Sentinel Surveillance

The ANC sentinel surveillance is the main source of national HIV and syphilis trend data in Zambia. Detailed descriptions of the methodology of the ANC sentinel surveillance system and developments over the period 1994–2008 have been published [13], [14], [17], [18], [19], [20]. In brief, this paper focuses on the 22 sites that have consistently been part of the surveillance system since 1994. The target sample of pregnant women was 500 in most sites, but in Ndola and the four sites in Lusaka (and in Livingstone and Kapiri Mposhi in 1994 and 1998) the target was 800. The focus for this paper was young women aged 15–24 years who participated in the surveys, giving a total sample of 39,064 (5542 in 1994, 7106 in 1998, 7092 in 2002, 6606 in 2004, 6575 in 2006 and 6143 in 2008). Women were enrolled consecutively in the study and socio-demographic data were collected through routine interviews. The data collection period was four months.

Blood specimens obtained from eligible women were tested for both syphilis and HIV in the local laboratory. The specimens for HIV testing were anonymous and unlinked to the women. Capillus HIV-1/HIV-2 (Cambridge Diagnosis Ltd., UK) was the first test for screening of HIV in the 1994–2002 surveys, and all reactive specimens were re-tested using a confirmatory test, Wellcozyme HIV (Murex Diagnosis Ltd., UK). Subsequently, the screening test was Abbot Determine HIV1/HIV2 (Abbot Laboratories, USA) and all reactive specimens were re-tested using Murex ICE HIV (Murex Diagnosis Ltd., UK). Discordant specimen results were re-tested using a tie-breaker, Bionor HIV1&2 (BIONOR AS, Norway). For quality control, 10% (5% each in 1994 and 1998) of the negative specimens and all positive specimens were re-tested using Abbot Determine. If one of the negative specimens was found positive, then an additional 40–50% of the negative results were re-tested. If more false negatives were discovered, then the entire bunch of negative specimens was re-tested. Two national reference laboratories performed the quality control and confirmatory testing: Tropical Diseases Research Centre (TDRC) in Ndola catered for the northern region of Zambia, and University Teaching Hospital Virology Laboratory in Lusaka catered for the southern region.

### Zambia Demographic and Health Surveys (ZDHS)

The ZDHS is a nationally representative population-based survey, and HIV testing was included in selected households in its 2001/2002 and 2007 rounds [21], [22], [23]. A two-stage cluster (proportional to size) sampling procedure was followed using the 2000 Population Census as the sampling frame. In the selected clusters, all households were listed and a systematic random sampling of households was undertaken. Women aged 15–49 years and men aged 15–59 years were eligible to participate in the survey. In the 2001/02 round, men were only eligible for interview in one-third of the selected households, and men and women in these households were asked to consent to HIV testing. In the 2007 survey, men in all selected households were eligible for interview and all eligible men and women who consented were tested for HIV infection. The general response rates to the interview were 95% (89% among men and 98% among women) and 94% (91% among men and 96% among women) in the 2001/02 and 2007 rounds, respectively. Of those eligible, 76% (2001/02) and 77% (2007) consented to the HIV test. The total sample of participants aged 15–24 years comprised 3146 males (669 in 2001/02 and 2477 in 2007) and 3960 females (957 in 2001/02 and 3003 in 2007). Other details have been presented elsewhere [21], [22], [23].

HIV testing in the 2001/2002 survey was anonymous, and only sex, age, province and urban/rural residence could be linked to the HIV data. Venous blood was collected on filter paper cards as dried blood spots (DBS) [21], [24]. However, in the 2007 survey, capillary blood from finger pricks was used for the DBS [22], and new protocols were followed that allowed HIV data to be linked to household and individual information while maintaining the anonymity of the participants. Wellcozyme HIV-1&2 GACELISA was used to test for HIV antibodies in the 2001/2002 survey. All positive specimens and 10% of the negative specimens were re-tested using Bionor HIV 1&2, and discordant specimens were tested with Western blot [21]. For the 2007 survey, the screening test was Vironostika HIV & Biomerieux, and all positive specimens were re-tested with a confirmatory test, Enzygnost Anti-HIV1/2 Plus (Dade Behring). Discordant specimens were tested using Western blot [22]. Ten percent of the specimens (both positive and negative) collected in both surveys were sent to the Global Clinical Viral Laboratory (GCVL) in Durban, South Africa for external quality assessment and the results had a 99% agreement rate with TDRC [21], [22], [23].

### Review of past literature

Published peer-reviewed articles on HIV prevalence and incidence in Zambia were identified through a computer search of the databases PubMed, Web-of-Science, EMBASE, Google-scholar, and African Journal on-line. The search was done with the following words: “HIV prevalence” OR “HIV incidence” OR “HIV trend” AND “Zambia”. Studies were selected on the basis of the following criteria: (1) must have been undertaken in Zambia, (2) must be an original publication of HIV data from a sample likely to be representative of the general population, and (3) must present HIV prevalence or incidence data. The search identified 22 articles that met the criteria and these were based on four data sources, apart from the ANC and DHS data.

*The Chelstone and Kapiri Mposhi population-based surveys (PBS)* were undertaken in the township of Chelstone in Lusaka and in rural Kapiri Mposhi district. Data were collected serially in 1995, 1999 and 2003. Random cluster sampling was used and all adults aged 15–49 years in all households in the selected clusters were eligible for the interview and the HIV test. Saliva samples were collected and tested for HIV using Gacelisa HIV 1&2 (in the 1995 survey) and Bionor HIV 1&2 (1999 and 2003 surveys). Further details of the survey methodology have been reported elsewhere [14], [15], [17], [20].

*The prevention of mother to child transmission (PMTCT)-surveys* were undertaken between 2002 and 2006. Their main objective was to examine trends in HIV seroprevalence among pregnant and parturient women in Lusaka district. Two data sources were used: (1) Routine data on number of women counselled and tested for HIV, number of women with positive test results, and number of women and infants on antiretroviral (ARV) prophylaxis from 24 public health facilities with PMTCT programmes (between July 2002 and December 2006); (2) Two surveillance rounds (June–August 2003 and October 2005–January 2006) with testing for maternal IgG HIV antibodies in umbilical cord-blood from 10,194 discarded placentas (from all facility-based live-births in Lusaka during the specified periods). Seropositivity of the umbilical-cord blood indicated maternal HIV infection. Specimens were tested for HIV using Determine HIV1&2. Full details of the study are provided elsewhere [25].

*The “Four cities” multicentre study* was conducted between 1997 and 1998 in four African countries; in Zambia, Ndola was selected. Two-stage random cluster sampling was used to select households in which all adults (15–49 years) were eligible for interview and blood specimen collection. The target sample size was 1000 men and 1000 women, of which 367 men and 510 women aged 15–29 years provided blood for HIV testing. Blood specimens were tested for HIV, syphilis and herpes simplex virus type two. The clusters were selected in the catchment areas of five ANC clinics, resulting in 40% of all women seen in these antenatal clinics being eligible for the population survey. In addition, 824 antenatal attendees aged 15–29 years were interviewed and tested for syphilis and HIV. The HIV test was performed using an enzyme-linked immunosorbent assay. Further details on the study have been published elsewhere [26], [27].

*The Microbicide clinical preparedness study* was a cohort study conducted in Chilenje and Kamwala health centres in Lusaka. The target population was women aged 16–49 years who were recruited through community meetings and local family planning meetings. All consenting women were screened for HIV and STIs and those found negative were enrolled into the study, giving a total of 239 women. These women were followed up for one year with monthly study visits (June 2003–October 2004). HIV incidence was estimated from venous blood specimens collected every three months. The testing was done using an ELISA test for screening and Western blot as a confirmatory test [28], [29].

### Statistical analysis

Analyses in this paper were restricted to young people aged 15–24 years [9], [10]. ANC and ZDHS data were analysed with SPSS 18 (IBM SPSS, Chicago, Illinois) and STATA Intercooled version 11.0 (StataCorp LP, College Station, Texas). We estimated HIV prevalence trends by individual ANC sites and socio-demographic characteristics using the *chi-square* (χ^2^) linear-by-linear trend test in SPSS. Trends by province and urban-rural residence (urban and rural were defined according to the Zambian Central Statistical Office standards) were examined by age-adjusted risk ratios (aRR) using *log-binomial regression of the generalized linear model* in STATA.

The direction and magnitude of changes in HIV prevalence among women were compared between 2002 and 2008 in the ANC data and between the ZDHS 2001/2 and 2007. Both data sources were stratified by age and province, and the changes in prevalence estimates were measured by age-adjusted risk ratios. Analyses of ZDHS data were adjusted for clustering and weighted to account for differential sampling and response probabilities. Prevalence changes for young men were also estimated, stratifying by urban/rural residence and age, but not by province since this yielded subgroups that were too small. Furthermore, we conducted a sensitivity analysis of the ZDHS estimates to assess the extent to which different scenarios of bias due to non-participation (refusals and absence) affected the prevalence estimate. We assessed three different scenarios, each assuming the HIV prevalence ratio to be somewhat higher among people who refused than among those who were absent in comparison with participants. The prevalence ratios tested were as follows: Scenario 1: Refusals/participations ratio 1∶3 and absentees/participants ratio 1∶1; the respective ratios in scenario 2 were 1∶5 and 1∶3; and in scenario 3, 2∶1 and 1∶5 (Table S1). The percentages of refusals and absences were obtained from the reports of the two surveys [21], [22].

### Ethics

The Ethics and Research sub-committee of the National AIDS Surveillance Committee approved the HIV sentinel surveillance in Zambia in 1989. Procedures were instituted to fulfil the requirements for unlinked anonymous testing of blood specimens collected as part of routine antenatal care. For the ZDHS, the Ethical Review Committee of the University of Zambia and the Institutional Review Board of ORC Macro of USA approved the protocols for the surveys. Informed consent was sought using oral and written methods from eligible participants and from the parent or guardian if the respondent was less than 18 years old. For HIV testing, separate consent was sought. The participants were not told their HIV results, but those who wanted to know their status were referred to VCT centres for counselling and testing. Details of ethical approvals for the other studies reviewed are provided elsewhere [15], [17], [20], [25], [26], [27], [28], [30].

## Results

### Trends in HIV prevalence at ANC surveillance sites

Overall, the five rounds of antenatal surveillance from 22 sites conducted during the period 1994–2008 show that HIV prevalence among young women aged 15–24 years has decreased substantially in both urban (median 27.4% to 15.5%) and rural (median 11.4% to 6.4%) sites. All urban sites except Kalingalinga and Matero had significantly falling trends; whereas among rural sites, only four (Macha, Isoka, Kasaba and Mukinge) had significantly decreasing trends. The rest of the rural sites showed no clear trend or had a non-significant increase in HIV prevalence (Kabompo) (Table 1).

**Table 1:** HIV prevalence among young people (15–24 year) for ANC sites with data from 1994 to 2008 by location of sites.

Urban								Rural							
	1994	1998	2002	2004	2006	2008	P-value		1994	1998	2002	2004	2006	2008	P-value
Site name	n	n	n	n	n	n		Site name	n	n	n	n	n	n	
	(%)	(%)	(%)	(%)	(%)	(%)			(%)	(%)	(%)	(%)	(%)	(%)	
Mongu	276	291	311	279	250	289		Kalabo	149	217	249	274	300	297	
	(30.1)	(27.1)	(30.2)	(23.3)	(14.8)	(25.6)	**0.005**		(8.1)	(13.4)	(14.5)	(14.2)	(9.7)	(12.1)	0.730
Livingstone	337	437	315	165	291	275		Macha	280	285	268	271	264	173	
	(32.0)	(29.5)	(29.8)	(27.9)	(21.6)	(22.2)	**0.001**		(7.9)	(7.0)	(6.3)	(5.9)	(3.4)	(1.2)	**0.001**
Chelstone	268	474	447	411	460	377		Kapiri Mp	284	498	312	239	244	226	
	(25.0)	(22.8)	(19.9)	(17.8)	(15.0)	(11.1)	**<0.001**		(13.4)	(16.5)	(22.8)	(20.1)	(9.4)	(17.7)	0.587
Chilenje	273	288	426	427	345	400		Minga	287	293	298	288	284	202	
	(34.8)	(21.9)	(26.8)	(18.0)	(19.1)	(15.8)	**<0.001**		(8.0)	(10.6)	(7.7)	(19.4)	(6.0)	(3.5)	0.537
Kalingalinga	280	286	338	403	427	404		Isoka	274	316	296	269	254	273	
	(20.0)	(23.4)	(20.1)	(19.9)	(19.7)	(15.1)	0.061		(11.3)	(10.1)	(7.1)	(11.5)	(3.1)	(5.1)	**0.001**
Matero	248	297	487	482	424	376		Nchelenge	262	301	283	274	274	257	
	(28.2)	(22.9)	(21.6)	(26.3)	(25.5)	(19.9)	0.195		(13.7)	(13.0)	(18.4)	(15.3)	(9.9)	(14.8)	0.906
Kabwe	275	306	280	263	277	254		Kasaba	261	276	158	205	142	167	
	(28.4)	(24.8)	(22.1)	(23.6)	(16.2)	(24.4)	**0.019**		(11.5)	(5.1)	(4.4)	(3.4)	(2.8)	(3.0)	**<0.001**
Chipata	261	289	283	280	290	268		Ibenga	218	263	223	217	175	179	
	(27.6)	(24.2)	(21.9)	(8.9)	(19.0)	(14.6)	**<0.001**		(11.0)	(8.0)	(8.1)	(7.8)	(10.3)	(7.3)	0.387
Kasama	251	308	299	306	282	280		Mukinge	205	206	281	250	269	143	
	(21.9)	(12.3)	(12.0)	(13.4)	(18.8)	(8.6)	**0.007**		(9.8)	(6.8)	(4.3)	(5.2)	(5.9)	(2.1)	**0.005**
Mansa	268	346	299	249	284	236		Kabompo	159	164	219	302	336	295	
	(23.5)	(20.8)	(21.4)	(24.5)	(15.1)	(16.9)	**0.037**		(1.9)	(9.8)	(5.9)	(7.9)	(5.4)	(8.8)	0.112
Ndola	288	612	578	450	394	475									
	(27.1)	(25.8)	(21.6)	(22.2)	(18.0)	(15.2)	**<0.001**								
Solwezi	120	295	310	302	289	297									
	(20.8)	(16.6)	(11.9)	(12.6)	(15.2)	(11.4)	**0.018**								
**Total**	**3145**	**4229**	**4373**	**4017**	**4013**	**3931**			**2379**	**2819**	**2587**	**2589**	**2542**	**2212**	
**(Mean)**	**(27.0)**	**(23.1)**	**(21.7)**	**(19.8)**	**(18.4)**	**(16.5)**	**<0.001**		**(10.0)**	**(10.6)**	**(10.4)**	**(11.3)**	**(6.6)**	**(8.3)**	**0.001**
**(Median)**	**(27.4)**	**(23.2)**	**(21.6)**	**(21.0)**	**(18.4)**	**(15.5)**			**(11.4)**	**(10.0)**	**(7.4)**	**(9.7)**	**(6.0)**	**(6.4)**	

*X^2^* linear trend tests. The highlighted p-values are statistically significant at 0.05 level. “n” is number, % is percent. Rural/urban refers to the location of the sites.

### HIV prevalence trends and relative risk ratios by urban-rural residence and province

Table 2 showed that HIV prevalence proportionally decreased in the five rounds of the ANC, by 39% among young urban women and by 27% among young rural women. Overall, HIV prevalence among urban women was almost twice that of rural women at baseline. However, significant and gradual declines in HIV prevalence trends were more prominent among young urban women than among young rural women. In fact, significant declines among rural residents occurred only between 2004 and 2008.

**Table 2:** ANC HIV prevalence and age-adjusted risk ratios for changes in HIV prevalence by province and residence (urban/rural) among young women 15–24 years.

	Year	Urban			Rural		
		*n*	%	aRR (95% CI)	*n*	%	aRR (95% CI)
**Total (22 Sites)**	1994	*2770*	*27.9*	1.00	*2754*	*11.5*	1.00
	1998	*3974*	*23.3*	**0.85 (0.78–0.92)**	*3042*	*11.3*	0.99 (0.86–1.14)
	2002	*4447*	*21.8*	**0.79 (0.73–0.86)**	*2471*	*10.1*	0.88 (0.76–1.04)
	2004	*3862*	*20.2*	**0.71 (0.65–0.77)**	*2744*	*11.3*	0.95 (0.82–1.11)
	2006	*3778*	*18.7*	**0.67 (0.61–0.73)**	*2677*	*7.0*	**0.60 (0.51–0.72)**
	2008	*3683*	*16.9*	**0.59 (0.54–0.65)**	*2381*	*8.3*	**0.72 (0.60–0.85)**
**Province**							
**Central (two sites)**	1994	*276*	*28.3*	1.00	*283*	*13.4*	1.00
	1998	*599*	*22.9*	0.83 (0.66–1.05)	*204*	*10.3*	0.77 (0.47–1.28)
	2002	*333*	*21.9*	0.80 (0.61–1.06)	*248*	*23.8*	**1.77 (1.22–2.57)**
	2004	*362*	*22.9*	0.83 (0.64–1.08)	*140*	*19.3*	1.41 (0.90–2.21)
	2006	*372*	*15.6*	**0.58 (0.43–0.78)**	*131*	*7.6*	0.57 (0.29–1.10)
	2008	*393*	*22.9*	0.82 (0.63–1.06)	*78*	*12.8*	0.95 (0.50–1.82)
**Copperbelt (two sites)**	1994	*337*	*24.0*	1.00	*169*	*12.4*	1.00
	1998	*606*	*26.1*	1.13 (0.90–1.42)	*268*	*7.5*	0.60 (0.34–1.08)
	2002	*557*	*22.4*	0.98 (0.77–1.25)	*242*	*7.4*	0.60 (0.33–1.09)
	2004	*446*	*22.2*	0.90 (0.70–1.17)	*221*	*8.1*	0.63 (0.35–1.15)
	2006	*384*	*18.0*	0.78 (0.58–1.03)	*178*	*9.6*	0.75 (0.41–1.37)
	2008	*458*	*15.3*	**0.63 (0.48–0.84)**	*188*	*8.0*	0.63 (0.33–1.18)
**Eastern (two sites)**	1994	*246*	*28.9*	1.00	*302*	*7.9*	1.00
	1998	*222*	*24.3*	0.91 (0.68–1.24)	*360*	*31.1*	**1.59 (1.00–2.53)**
	2002	*271*	*22.5*	0.85 (0.63–1.13)	*309*	*7.8*	0.96 (0.56–1.65)
	2004	*287*	*19.5*	**0.70 (0.51–0.94)**	*281*	*8.9*	1.08 (0.63–1.83)
	2006	*268*	*18.7*	**0.68 (0.50–0.93)**	*295*	*6.1*	0.72 (0.40–1.28)
	2008	*241*	*13.3*	**0.46 (0.32–0.67)**	*224*	*6.3*	0.76 (0.40–1.43)
**Luapula (three sites)**	1994	*60*	*23.3*	1.00	*731*	*15.7*	1.00
	1998	*132*	*22.0*	0.99 (0.56–1.72)	*787*	*12.2*	0.79 (0.62–1.02)
	2002	*254*	*24.0*	1.11 (0.67–1.84)	*484*	*12.8*	0.86 (0.65–1.14)
	2004	*238*	*24.4*	1.03 (0.62–1.72)	*490*	*10.6*	**0.65 (0.48–0.89)**
	2006	*125*	*17.6*	0.78 (0.43–1.41)	*557*	*9.0*	**0.55 (0.40**–**0.75)**
	2008	*81*	*13.6*	0.63 (0.31–1.28)	*562*	*12.1*	**0.75 (0.57**–**0.99)**
**Lusaka (four sites)** β	1994	*1032*	*27.6*	1.00			
	1998	*1182*	*22.6*	**0.84 (0.73–0.97)**			
	2002	*1579*	*22.2*	**0.80 (0.70–0.92)**			
	2004	*1561*	*20.9*	**0.74 (0.64–0.85)**			
	2006	*1534*	*20.2*	**0.72 (0.63–0.83)**			
	2008	*1522*	*15.7*	**0.54 (0.47–0.63)**			
**Northern (two sites)**	1994	*142*	*29.6*	1.00	*383*	*11.5*	1.00
	1998	*243*	*14.4*	**0.51 (0.34–0.75)**	*380*	*9.2*	0.77 (0.51–1.17)
	2002	*359*	*13.6*	**0.49 (0.34–0.70)**	*234*	*3.4*	**0.29 (0.14–0.61)**
	2004	*234*	*13.2*	**0.48 (0.31–0.72)**	*341*	*12.0*	0.98 (0.66–1.46)
	2006	*211*	*20.4*	**0.69 (0.48–0.99)**	*321*	*5.6*	**0.46 (0.27–0.78)**
	2008	*162*	*9.3*	**0.33 (0.19–0.57)**	*384*	*5.7*	**0.48 (0.29–0.78)**
**North-Western (three sites)**	1994	*101*	*22.8*	1.00	*383*	*6.5*	1.00
	1998	*315*	*16.8*	0.75 (0.49–1.14)	*341*	*7.3*	1.14 (0.67–1.94)
	2002	*378*	*11.6*	**0.57 (0.36–0.89)**	*429*	*4.2*	0.66 (0.36–1.18)
	2004	*396*	*10.4*	**0.46 (0.29–0.72)**	*458*	*7.4*	1.14 (0.69–1.88)
	2006	*321*	*15.3*	0.67 (0.43–1.04)	*558*	*5.0*	0.79 (0.46–1.33)
	2008	*300*	*12.0*	**0.54 (0.34–0.86)**	*432*	*6.3*	0.98 (0.58–1.66)
**Southern (two sites)**	1994	*327*	*32.1*	1.00	*290*	*8.6*	1.00
	1998	*413*	*30.0*	0.95 (0.77–1.17)	*305*	*8.2*	0.96 (0.57–1.63)
	2002	*301*	*30.6*	0.94 (0.75–1.18)	*277*	*6.5*	0.77 (0.43–1.38)
	2004	*161*	*28.6*	0.86 (0.65–1.14)	*275*	*5.8*	0.67 (0.36–1.22)
	2006	*273*	*21.6*	**0.67 (0.51–0.88)**	*281*	*4.6*	0.55 (0.29–1.06)
	2008	*271*	*22.1*	**0.69 (0.53–0.90)**	*166*	*1.2*	**0.14 (0.03–0.59)**
**Western (two sites)**	1994	*249*	*29.7*	1.00	*176*	*11.9*	1.00
	1998	*262*	*26.7*	0.90 (0.69–1.18)	*245*	*15.1*	1.23 (0.75–2.02)
	2002	*415*	*27.2*	0.92 (0.72–1.17)	*142*	*12.0*	1.01 (0.56–1.84)
	2004	*177*	*22.0*	0.72 (0.52–1.00)	*376*	*17.3*	1.39 (0.88–2.19)
	2006	*290*	*15.9*	**0.53 (0.38–0.73)**	*256*	*7.8*	0.65 (0.36–1.16)
	2008	*255*	*26.7*	0.89 (0.68–1.17)	*321*	*12.1*	1.02 (0.62–1.67)

β A total of 600 women recorded as rural residents attending ANC sites in Lusaka were excluded from the analysis because all the sites in Lusaka were urban and Lusaka is predominantly an urban district. Age adjustment was done using a continuous age variable. The highlighted p-values are statistically significant at 0.05 level, n is number, % is percentage, aRR is age-adjusted risk ratio.

At provincial level, the most consistent and significant declines were among young urban women in Lusaka, Northern and North-Western provinces. Further, urban residents of Eastern province and rural residents of Luapula province had significant declines between the 2002 and the 2008 surveys, whereas in Southern (urban women) and Northern provinces (rural women) there were consistent and substantial declines in the period between 2004 and 2008. Otherwise, most provinces showed either a stable prevalence or fluctuations in prevalence over time (Table 2).

### ANC data vs. ZDHS data

While significant declines were observed among both urban and rural women in the ANC surveys, the decreases observed in the ZDHS among urban and rural young women were non-significant. A comparison of pregnant women and women in the general population showed that the directions and magnitudes of change in the period evaluated were largely similar (30% vs. 22% among urban residents and 20% vs. 22% among rural residents, respectively). Despite a tendency towards decline in HIV prevalence among urban ANC participants in most provinces, there were no significant changes at province level for young urban female ZDHS participants. The provincial level changes for rural ANC participants and female ZDHS participants were mostly non-significant (Table 3). There were a non-significant increase in HIV prevalence among urban men aged 15–24 years (from 3.7% to 5.0%, aRR 1.67 CI 0.75–3.69) and among younger rural men aged 15–19 years (from 2.6% to 3.0%, aRR 1.86 CI 0.75–4.64).

**Table 3:** HIV prevalence and age-adjusted risk ratios estimates for ANC (2002 and 2008) and ZDHS (2001–2002 and 2007) by age-group and province.

	ANC			ZDHS					
	Women			Women			Men		
	2002	2008		2001/2002	2007		2001/2002	2007	
	%	%	aRR (95% CI)	%	%	aRR (95% CI)	%	%	aRR (95% CI)
**Urban**	*21.8*	*16.8*	**0.72 (0.66–0.80)**	*15.2*	*12.5*	0.79 (0.58–1.08)	*3.7*	*5.0*	1.67 (0.75–3.69)
**Age-Group**									
**15–19**	*16.6*	*11.7*	**0.70 (0.58–0.85)**	*9.1*	*6.5*	0.61 (0.34–1.12)	*1.9*	*3.5*	1.78 (0.41–7.65)
**20–24**	*25.3*	*19.4*	**0.76 (0.69–0.85)**	*22.4*	*19.8*	0.82 (0.57–1.19)	*5.4*	*7.2*	1.57 (0.60–4.08)
**Province**									
**Central**	*21.9*	*22.9*	1.03 (0.78–1.35)	*15.9*	*15.3*	1.09 (0.47–2.51)	*4.3*	*10.4*	2.41 (0.32–18.0)
**Copperbelt**	*22.4*	*15.3*	**0.64 (0.49–0.84)**	*12.3*	*8.9*	0.76 (0.40–1.44)	*2.8*	*5.2*	1.90 (0.42–8.52)
**Eastern**	*22.5*	*13.3*	**0.53 (0.36–0.78)**	*0.0*	*11.1*	-	*0.0*	*2.9*	-
**Luapula**	*24.0*	*13.6*	**0.56 (0.32–1.01)**	*11.1*	*10.5*	0.84 (0.17–4.23)	*4.2*	*2.2*	-
**Lusaka**	*22.2*	*15.7*	**0.68 (0.58–0.78)**	*20.8*	*10.8*	0.63 (0.37–1.07)	*0.0*	*1.6*	-
**Northern**	*13.6*	*9.3*	0.68 (0.40–1.17)	*11.8*	*8.8*	0.66 (0.16–2.79)	*6.0*	*7.3*	-
**N/Western**	*11.6*	*12.0*	0.93 (0.62–1.40)	*5.9*	*9.4*	1.50 (0.20–11.2)	*0.0*	*1.0*	-
**Southern**	*30.6*	*22.1*	**0.73 (0.55–0.96)**	*21.7*	*19.7*	0.94 (0.40–2.19)	*0.0*	*5.2*	-
**Western**	*27.2*	*26.7*	0.97 (0.76–1.25)	*18.2*	*21.1*	1.07 (0.34–3.33)	*16.7*	*9.4*	0.25 (0.02–2.68)
									
**Rural**	*10.0*	*8.3*	**0.80 (0.67–0.96)**	*7.8*	*6.4*	0.74 (0.52–1.07)	*3.1*	*2.9*	1.12 (0.57–2.22)
**Age-Group**									
**15–19**	*7.7*	*5.9*	0.76 (0.55–1.06)	*4.3*	*6.0*	1.26 (0.68–2.33)	*2.6*	*3.0*	1.86 (0.75–4.64)
**20–24**	*12.0*	*9.9*	0.83 (0.67–1.02)	*11.6*	*6.8*	**0.55 (0.34–0.87)**	*3.9*	*2.8*	0.72 (0.28–1.89)
**Province**									
**Central**	*23.8*	*12.8*	**0.54 (0.29–0.99)**	*7.5*	*15.0*	1.75 (0.72–4.25)	*3.4*	*3.0*	0.91 (0.19–4.39)
**Copperbelt**	*7.4*	*8.0*	1.01 (0.53–1.96)	*11.1*	*4.1*	**0.15 (0.04–0.57)**	*0.0*	*2.0*	-
**Eastern**	*7.8*	*6.3*	0.80 (0.42–1.50)	*10.4*	*2.9*	**0.31 (0.11–0.86)**	*4.2*	*2.2*	0.41 (0.09–1.88)
**Luapula**	*12.8*	*12.1*	0.86 (0.62–1.18)	*6.5*	*8.2*	1.29 (0.43–3.83)	*0.0*	*12.0*	-
**Lusaka** β	*-*	*-*	-	*5.0*	*8.3*	1.13 (0.18–7.01)	*0.0*	*2.2*	-
**Northern**	*3.4*	*5.7*	1.58 (0.72–3.48)	*5.5*	*3.9*	0.65 (0.22–1.90)	*1.1*	*2.4*	2.10 (0.22–19.9)
**N/Western**	*4.2*	*6.3*	1.49 (0.83–2.66)	*6.5*	*3.1*	0.48 (0.13–1.73)	*6.6*	*0.0*	**-**
**Southern**	*6.5*	*1.2*	**0.18 (0.04–0.77)**	*6.9*	*6.5*	0.93 (0.33–2.61)	*3.8*	*2.6*	0.72 (015–3.50)
**Western**	*12.0*	*12.1*	1.01 (0.60–1.73)	*12.7*	*8.4*	0.65 (0.28–1.49)	*2.4*	*1.1*	0.55 (0.03–11.4)

β A total of 600 women recorded as rural residents attending ANC sites in Lusaka were excluded from the analysis because all the sites in Lusaka were urban and Lusaka is predominantly an urban district. The highlighted p-values are statistically significant at 0.05 level, n is number and % is percentage, aRR is age- adjusted risk ratio. The dash (–) represent missing cases.

### HIV prevalence trends and socio-demographic characteristics

**Figure 1:**
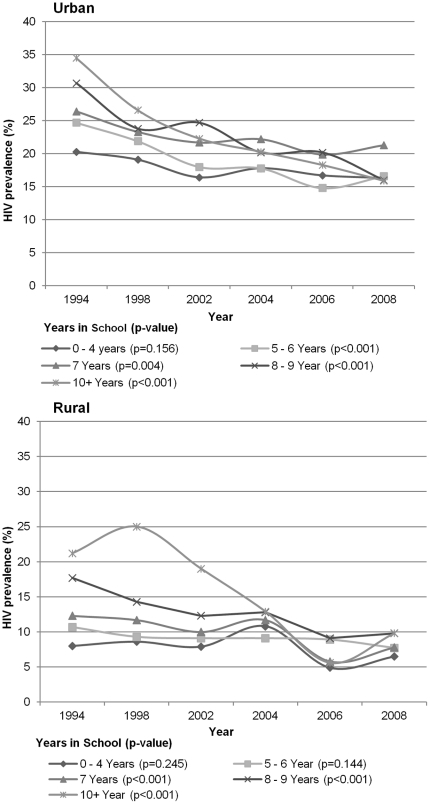
ANC-based trends in HIV prevalence by educational attainment 1994–2008.

ANC data further indicated a significant decrease in HIV prevalence irrespective of marital status. Among both urban and rural married women, significantly declining HIV prevalence trends were observed (from 27.8% to 17.1% and from 11.6% to 7.4%, respectively, p<0.001). Similar declines were observed among urban single women (27.1% to 15.6%, p<0.001) and rural (10.7% to 9.4%, p = 0.030). Furthermore, clearly marked declines were also seen among young urban residents with more than five years of schooling and among young rural residents with more than six years of schooling (p<0.001) (Figure 1). HIV prevalence has been consistently lower among less educated young women than among those with more education, but only a non-significant decline occurred during the five rounds of the ANC in both urban (p = 0.159) and rural (p = 0.245) areas.

### ANC data vs. other surveys in Zambia

There is a general consensus among the trend studies that HIV prevalence among young women is decreasing despite the use of different methods and different populations to estimate the prevalence. The population-based surveys from Chelstone showed a 44% relative decrease in HIV prevalence among young participants aged 15–24 years in the three rounds of the study (from 16.5% to 8.5%), whereas among young ANC-attendees in Chelstone the prevalence decreased proportionally by 20% between 1994 and 2002 (from 25.0% to 19.9%) [3]. In the same Chelstone study, it was found that HIV prevalence among less educated young men and women was stable, but among the educated young people, irrespective of gender, the trends decreased significantly. During the same period, there was no clear trend among young educated ANC attendees and a non-significant decrease for the less educated ones. The HIV prevalence among women (15–24 years) attending PMTCT-sites in Lusaka decreased steadily from 24.8% (2002) to 21.6% (2006) [25]. Within the same study, the umbilical cord blood surveillance rounds also showed a reduction in HIV prevalence among Lusaka women under the age of 25 years from 21.7% in 2003 to 17.3% in 2005/06 [25]. Further comparisons revealed that HIV prevalence in the four Lusaka ANC surveillance sites (Chelstone, Chilenje, Kalingalinga and Matero) decreased from 22.2% in 2002 to 20.2% in 2006.The “Microbicide preparedness study” found the rate of new infections among young women (18–24 years) in Chilenje and Kamwala residential areas in Lusaka to be 4.7 per 100 person-years [28], [29].

In the Ndola part of the ‘Four cities’ multicentre study conducted in 1997/1998, the crude HIV prevalence estimates for youths aged 15–29 years in the general population was 28.3%, (15.0% for men and 37.8% for women), whereas the ANC-data in Ndola during the same year for young females (15–29 years) gave an estimate of 28.2% (Table 4).

**Table 4:** HIV prevalence and incidence among young women and men aged 15–24 years from surveys in Zambia by residence between 1994 and 2008.

Residence	Type of Survey and location	Year	Crude HIV Prevalence (%)		
			Women	Men	ANC prevalence estimates from corresponding geographical area
**Urban**	Chelstone PBS	1995	22.5	6.9	25.0
		1999	18.3	7.4	22.8
		2003	12.5	3.2	19.9
					
	Ndola PBS**	1997/98	37.8	15.0	28.2
					
	PMTCT Lusaka	2002	24.8		22.1
		2003	24.0		
		2004	23.0		20.7
		2005	22.3		
		2006	21.6		19.7
					
	Cord-blood Surveillance Lusaka	2003	21.7		
		2005/06	17.3		19.7
					
	Microbicide HIV incidence#	2003/04	47		
					
					
**Rural**	Kapiri Mposhi PBS	1995	16.1	5.7	13.4
		1999	10.3	7.5	16.5
		2003	6.8	3.2	22.8

HIV prevalence estimated based on assumed stable incidence between age 15 and 24.

** Ndola PBS age range used is 15–29 years.

# HIV Incidence was measured per 1000 person years. Blank spaces - no HIV data

The three population-based surveys in Kapiri Mposhi found a proportional decline in the HIV prevalence of 58% (from 16.1% to 6.8%; P<0.001) among young females and a non-significant decline among young men (from 5.7% to 3.2%; P = 0.143). HIV prevalence declines were more marked among educated men and women than among less educated ones in Kapiri Mposhi. In contrast, the ANC data from Kapiri Mposhi generally showed that within the same period (1994, 1998, 2002) there was an increase in HIV prevalence, as shown in table 4.

## Discussion

The different data sources and methods used to estimate HIV prevalence trends indicated overall falling HIV prevalence among young women in Zambia but also highlighted geographical differences. At provincial level, the ANC data showed substantial geographical variations in the prevalence trends, with declines ranging from between 10% and 68% among young urban women, and from stability in three provinces to 86% decline among young rural women. Although the point prevalence tended to differ between ANC data and population-based data sources, the overall results of the ANC were in agreement with the changes observed between the two population-based ZDHS. Furthermore, the more educated young pregnant women had substantial falling prevalence trends, whereas the less educated had almost stable HIV prevalence. Similar results were reported in the population-based studies in Chelstone and Kapiri Mposhi [15], [30].

In our study we used HIV prevalence estimates among young women aged 15–24 years as an indicator of HIV incidence. The only study that has attempted to estimate incidence in Zambia directly was the “Microbicide clinical preparedness study”, which drew its sample population from two communities in Lusaka [28]. The incidence estimates from this study (47 per 1000 person years) was much higher than national incidence estimates derived from mathematical modelling of pooled urban and rural ZDHS data (approximately 17 per 1000 and 12 per 1000 person years among 15–19 and 20–24 year old women, respectively) [31]. This difference is obviously due in part to the participation and selection criteria of participants, or to the differences in incidence between urban and rural women. A comparison with the HIV prevalence estimates from ANC surveillance and from the other studies conducted in Lusaka thus indicates that women recruited for the cohort study may have had a higher incidence than women in the ANC surveillance or the general population. This was expected since only sexually active women were included, and probably women who joined the microbicide study were more likely to perceive themselves as being at a heightened risk of HIV infection.

Our study also showed that declines in HIV prevalence varied by urban-rural residence and educational attainment. The ANC data indicated that HIV prevalence decline among young urban women in Zambia had started by the mid-1990s, whereas declines among young rural women became clearly evident only after 2004. The later change among rural residents may reflect differences in intensity and outreach of prevention campaigns in rural versus urban areas. For both urban and rural young women with more than seven years of educational attainment, a sharp drop in HIV prevalence can be observed from the mid or late 1990s. These results are in line with other studies on the association between HIV prevalence and educational attainment in sub-Saharan Africa [32], [33], including Zambia [3], [15]. In addition, a similar pattern of marked decrease in syphilis prevalence among educated women was observed in the same ANC data [34]. A likely explanation for this change is that educated people, once equipped with knowledge about HIV from prevention campaigns, have been quicker in changing their sexual behaviour [35], [36], [37], [38], [39], [40], [41].

Provincial ANC data suggest substantial inter-provincial differences in magnitude and trends of HIV prevalence. Most provinces in Zambia had either a stable or a decreasing HIV prevalence among both urban and rural residents. Lusaka, Northern and North-Western provinces recorded consistent and significant declines in HIV prevalence among urban ANC attendees during the period. Multiple factors are likely to explain the geographical differentials in magnitude and trends of HIV infection in Zambia. Variation in coverage and intensity of preventive efforts is an example. Furthermore, cultural and socio-economic factors are likely to have contributed substantially [16], [37]. For example, the interplay of structural and individual factors in Lusaka (i.e. relatively higher intensity of HIV preventive programmes and educational attainment) may have fostered sexual behaviour change, resulting in the decline reported here. Better understanding of the factors underlining these differentials in magnitude and trends might be critical for proper guidance of future preventive efforts.

Comparison of ANC and ZDHS data reveals that the provincial estimates for young urban women were similar in terms of direction (except in three provinces) but slightly different in terms of magnitude of change. Among young rural women, the provincial estimates of change differed both in direction and magnitude. It is likely that the differences seen were partially attributable to the small sample sizes of young people at provincial level in the ZDHS, since the provincial estimates were at least more in line with the magnitude and direction of change among urban women aged 15–49 years. HIV prevalence trends for men and women tend to be parallel [42], as seen in the population-based trends in selected urban and rural population [30] and other data sources, indicating that sexual risk taking among men in Zambia has declined during the same period (paper in progress); we believe that the apparent increase in HIV prevalence among young men in the ZDHS is also likely to be an artefact due to small sample sizes. However, if another round of the DHS shows differences in trend estimates for men and women, improved HIV surveillance of men should be considered.

In the Ndola data, the ANC-based estimate was similar to the crude HIV estimate of the overall general population but it underestimated the prevalence of females aged 15–29 years by 25%, possibly indicating that HIV infection may affect fertility at a young age [26]. Another factor influencing fertility is contraceptive use. Modern contraceptive use among women in Zambia's general population has increased gradually from 8.9% in 1992, to 14.4% in 1996, to 22.6% in 2001/02 and to 32.7% in 2007 [22]. In a situation where use of contraceptives in the general population is very high, the use of HIV prevalence of young women as a proxy of incidence can be less reliable because women may be sexually active for many years without becoming pregnant, leading to selection biases in the antenatal surveillance data. Hormonal contraceptive use has been linked to increased risk of HIV infection [43]. However, those who take contraceptives to prevent pregnancy might also take other precautions that put them at lower risk of HIV (e.g., condom use or having fewer sexual partners) than those who are sexually active and become pregnant. In line with this, educated women have been found to be both more likely to use contraceptives [22], [35] and to postpone sexual debut, leading to a strong association between educational attainment and reduced fertility [35]. Since HIV prevalence declines were biased towards higher educational attainment in Chelstone, postponement of first pregnancy among women with high education seems the most plausible explanation for the finding that ANC-based HIV prevalence trends substantially underestimated the actual declines in HIV prevalence in the general population [3]. The prevalence declines were also clearly biased towards higher education groups in the national ANC-based data presented here, so it is likely that the presented estimates underestimate actual trends in the population. Another potential bias affecting the trend estimates at ANC site level is changes in the coverage of rural clinics over time, leading to fluctuations in the number of urban and rural residents included at individual sites. Such changes in the outreach to rural residents are likely to explain the discrepancy observed in Kapiri Mposhi between the trend estimates obtained from ANC and population-based data [13].

Furthermore, we could not rule out the possibility that non-participating respondents or excluded non-household populations had a different risk of HIV infection from those who participated in the DHS. The sensitivity analyses showed that point prevalence estimates only increased by 1–2 percentage points in the most extreme scenario and that the magnitude of change between the 2001/02 and 2007 ZDHS was similar. We assumed that people who were absent had a lower risk of HIV than those who refused, and this is reasonable since data from the population-based surveys in Kapiri Mposhi and Chelstone indicated that the most common reason for absence among young people was school attendance. Being in school tends to be protective against HIV infection [17], [30]. Our sensitivity analysis is admittedly less sophisticated than that conducted by Bärnighausen et al using a Heckman-type selection model on the ZDHS 2007 data [44]. However, the estimates calculated in the latter paper indicate a prevalence ratio of 3∶7 among non-responders to participants, and this is highly unlikely. Our assumptions and results are likely to be more plausible.

Using PMTCT data to estimate HIV prevalence has inherent biases since women agreeing to participate in the PMTCT programme may be different from those refusing to participate [10]. The differences may relate to the risk of infection and the quality of counselling in the programme. However, a study conducted in Uganda found that this bias was only important in the initial period of PMTCT; after a couple of months there was no significant difference between accepting and refusing women [45]. This is consistent with the findings of the PMTCT study in Lusaka, since the acceptance rate for women attending ANC in the PMTCT sites increased (from 71% to 94% between July 2002 to December 2006) [25]. Furthermore, the PMTCT- and ANC-based estimates from Lusaka were closer in 2006 than 2002. It has been suggested that in situations where ANC and PMTCT coverage are very high and routinely collected PMTCT data are complete and accurate, routine PMTCT may replace the ANC surveillance system [46]. However, in most of sub-Saharan Africa, PMTCT data are still of poor quality [46], [47].

Bias in HIV surveys may also arise as a result of the antiretroviral therapy (ART) programme, stigma and migration. Scaling-up of the ART programme to most parts of Zambia has resulted in increased survival time among those accessing the therapy, and this might in the long run have implications for the reliability of using HIV prevalence among young people to estimate incidence. This is because HIV-positive children may survive into their teens [48] and become pregnant [49], thus distorting the assumption of recent infections among young people. However, since the ART programme was only implemented nationally in 2003 in Zambia, no such effect could yet be seen in 2008. HIV-related stigma could increase participation bias because some people may fear that others will discover their HIV status [50], [51]. Selection bias due to migration is possible in HIV trend studies since migrants who are at high risk of infection are less likely to participate, and this warrants further investigation [52].

### Conclusion

In conclusion, the findings suggest that although there are convincing HIV incidence declines in Zambia, the overall prevalence trend estimates have masked differential trends by place and by educational attainment. This might not only suggest differential and dynamic sub-population epidemics but also the need for tailored prevention programmes. Focusing on country-level trends in epidemiological reports therefore seems to have critical limitations and may even be directly misleading for policy makers and local programme managers who should base their efforts on comprehensive knowledge of the different epidemiological contexts within Zambia.

## Supporting Information

Table S1 **Sensitivity Analysis.**
(XLS)
